# Evaluating the Impact of Age and Sex on Survival in Localised Anterior Uveal Melanoma Undergoing Eye-Preserving Interventions Within a Surveillance, Epidemiology, and End Results (SEER) Cohort

**DOI:** 10.7759/cureus.93835

**Published:** 2025-10-04

**Authors:** Michael Milad, Mohammad Almanasra, Mohammad Al-khazaleh, Abraham Gabriel, Sarankan Sriranganathan

**Affiliations:** 1 Accident and Emergency, West Hertfordshire NHS Trust, Watford, GBR; 2 Oncology, Mount Vernon Cancer Center, East and North Hertfordshire NHS Trust, London, GBR; 3 Accident and Emergency, Stepping Hill Hospital, Stockport NHS Foundation Trust, Manchester, GBR; 4 Medicine, Ashford and St. Peter’s Hospitals NHS Foundation Trust, Chertsey, GBR; 5 Urology, East and North Hertfordshire NHS Trust, Stevenage, GBR

**Keywords:** ciliary body melanoma, iris melanoma, prognostic factors, survival analysis, uveal melanoma

## Abstract

Purpose

The purpose of the study was to evaluate the prognostic influence of age and sex on survival in patients with iris and ciliary body melanoma who were treated with eye-preserving interventions.

Methods

A retrospective cohort study was conducted using the Surveillance, Epidemiology, and End Results (SEER) database. We included patients diagnosed between 2000 and 2017 with localised iris or ciliary body melanoma who underwent local excision or radiotherapy. Kaplan-Meier curves with log-rank testing were used to assess survival differences by age (<50, 50-69, ≥70 years) and sex. We performed multivariable Cox regression to adjust for covariates.

Results

Ninety-nine patients met the inclusion criteria, with a median follow-up of 117 months. Age distribution was <50 years (n=17), 50-69 years (n=58), and ≥70 years (n=24). Overall survival significantly differed by age (log-rank p<0.001). Patients who were 70 years or older had more than a tenfold increased mortality risk compared with those who were younger than 50 (HR 10.65, 95% CI 2.46-46.07, p=0.002). The 50-69 years group demonstrated a trend toward increased risk (HR 3.99, 95% CI 0.94-17.01, p=0.061). In contrast, sex was not associated with survival (HR 1.02, 95% CI 0.54-1.92, p=0.944; log-rank p=0.87).

Conclusions

Age is a strong prognostic factor in localised iris and ciliary body melanoma treated with eye-preserving strategies, whilst sex does not influence survival. These findings underscore the value of demographic risk stratification in clinical management of anterior uveal melanoma.

## Introduction

Uveal melanoma is the most common primary intraocular malignancy in adults, although the vast majority arise in the choroid. In contrast, melanomas of the iris and ciliary body are rare, representing less than 10% of all uveal melanoma cases [1]. These tumours are often detected at an earlier stage compared to posterior uveal melanomas, largely because they may be visible on slit-lamp examination or cause ocular symptoms such as pigmented lesions, secondary glaucoma, or visual disturbance. Although the prognosis is generally more favourable than that of choroidal melanoma, a substantial subset of patients still develop metastasis, impacting survival [1]. 

Eye-preserving treatment, such as plaque brachytherapy, proton beam radiotherapy, or limited surgical excision, is the preferred approach in localised disease [2,3]. Advances in these modalities have enabled high local control whilst preserving vision, making evaluation of prognostic factors particularly relevant.

Age and sex are established prognostic factors across many cancers, including posterior uveal melanoma. Older age is linked to worse outcomes, whilst sex differences may reflect immune and hormonal influences [4,5]. Prior registry studies suggest that men and older patients have inferior survival, whilst ciliary body involvement independently predicts poorer prognosis [6]. Whilst prognostic determinants in posterior uveal melanoma have been studied extensively, the anterior subgroup has received far less attention. Understanding the demographic risk in this cohort is essential to guide counselling. 

Epidemiological data demonstrates a stable incidence of uveal melanoma, at approximately five cases per million annually. However, Surveillance, Epidemiology, and End Results (SEER) data from 1975-2020 demonstrates declining enucleation and an increased use of radiation [7]. Despite this, survival rates have not improved significantly. In contrast, Swedish population-based studies suggest that survival has improved in recent decades, possibly due to earlier detection and a wider use of brachytherapy [8]. Given the limited evidence regarding the prognostic impact of age and sex on iris and ciliary body melanomas specifically treated with eye-preserving strategies, our study addresses an important knowledge gap.

## Materials and methods

Data source and cohort selection

We performed a retrospective cohort study using the Surveillance, Epidemiology, and End Results (SEER) database of the United States National Cancer Institute (https://seer.cancer.gov), which is publicly and freely available. SEER is a population-based registry that contains information on cancer incidence, demographics, treatment, and survival for approximately one-third of the US population. The database is widely used for outcomes research due to its standardised data collection and rigorous quality controls. For this analysis, we identified cases from the SEER 17 registries [9]. Data were extracted using the SEER*Stat Software (version 8.4.2), which is publicly available [10].

Study cohort and eligibility criteria

Patients diagnosed between 2000 and 2017 with malignant melanoma of the uveal tract were identified using the International Classification of Diseases for Oncology, Third Edition (ICD-O-3) histology codes 8720-8799 [11]. In order to isolate anterior uveal melanomas, we only selected cases with the primary site recorded as in the ciliary body (site code C69.4). In SEER, this category included both iris and ciliary body melanomas. 

To ensure a uniform cohort, the analysis was restricted to cases of localised disease (the tumour confined to the eye, according to SEER historic stage A), and where the tumour represented the first and only primary malignancy [12]. Patients who underwent eye-preserving treatment were included. This included local surgical excision (SEER surgery codes 20-22 and 25, corresponding to partial excision, iridectomy, or iridotomy), and definitive beam radiation therapy without enucleation. We excluded patients who underwent chemotherapy, given that it is rare in this context, and could potentially introduce heterogeneity. Cases where there were incomplete demographic or survival data were also excluded. 

Both the ICD-O-3 histology and site codes, and the SEER historic stage are standardised, publicly available coding systems [11,12].

Variables

The primary demographic variables were patient age and sex. Age at diagnosis was categorised into three relevant groups: <50 years, 50-69 years, and ≥70 years. This is consistent with prior SEER-based uveal melanoma studies to allow comparability across cohorts and ensure adequate subgroup numbers for the survival analysis. As per the SEER coding, sex was recorded as male or female. We obtained tumour characteristics, year of diagnosis, and vital status directly from the database.

Outcome definition

The primary outcome was overall survival (OS), defined as the interval from diagnosis to death from any cause, or the last date of follow-up, whichever occurred first. Patients alive at the last follow-up were censored. Median follow-up time was calculated using the reverse Kaplan-Meier method.

Statistical analysis

Descriptive analysis was used to summarise patient demographics and tumour characteristics. We used the Kaplan-Meier method to estimate survival probabilities [13]. The log-rank test was used to compare survival distributions between subgroups, including age and sex [14].

Both univariate and multivariable survival analyses were performed. Multivariable Cox proportional hazards regression was used to examine the association of age and sex with overall survival [15]. These were expressed as hazard ratios (HRs), with 95% confidence intervals (CIs).

All statistical tests were performed in R (version 4.5.1; R Foundation for Statistical Computing, Vienna, Austria) [16]. Survival analyses were conducted using the survival package, whilst Kaplan-Meier curves were generated with the survminer package [17,18]. Both R and these packages are open-source and free to use.

All statistical tests were two-sided, and a p-value of <0.05 was considered statistically significant.

This study was based on de-identified, publicly available data from the SEER database; therefore, institutional review board approval and informed consent were not required.

## Results

We identified 99 patients with localised iris or ciliary body melanoma for analysis. The median follow-up period was 117 months (range: 1-274 months), during which 43 deaths occurred. Females comprised most of the cohort (61 patients, 61.6%), with 38 males (38.4%). Regarding age distribution, there were 17 patients (17.2%) younger than 50 years, 58 (58.6%) who were between 50-69 years, and 24 (24.2%) were aged ≥ 70 years.

Age and survival

**Figure 1:**
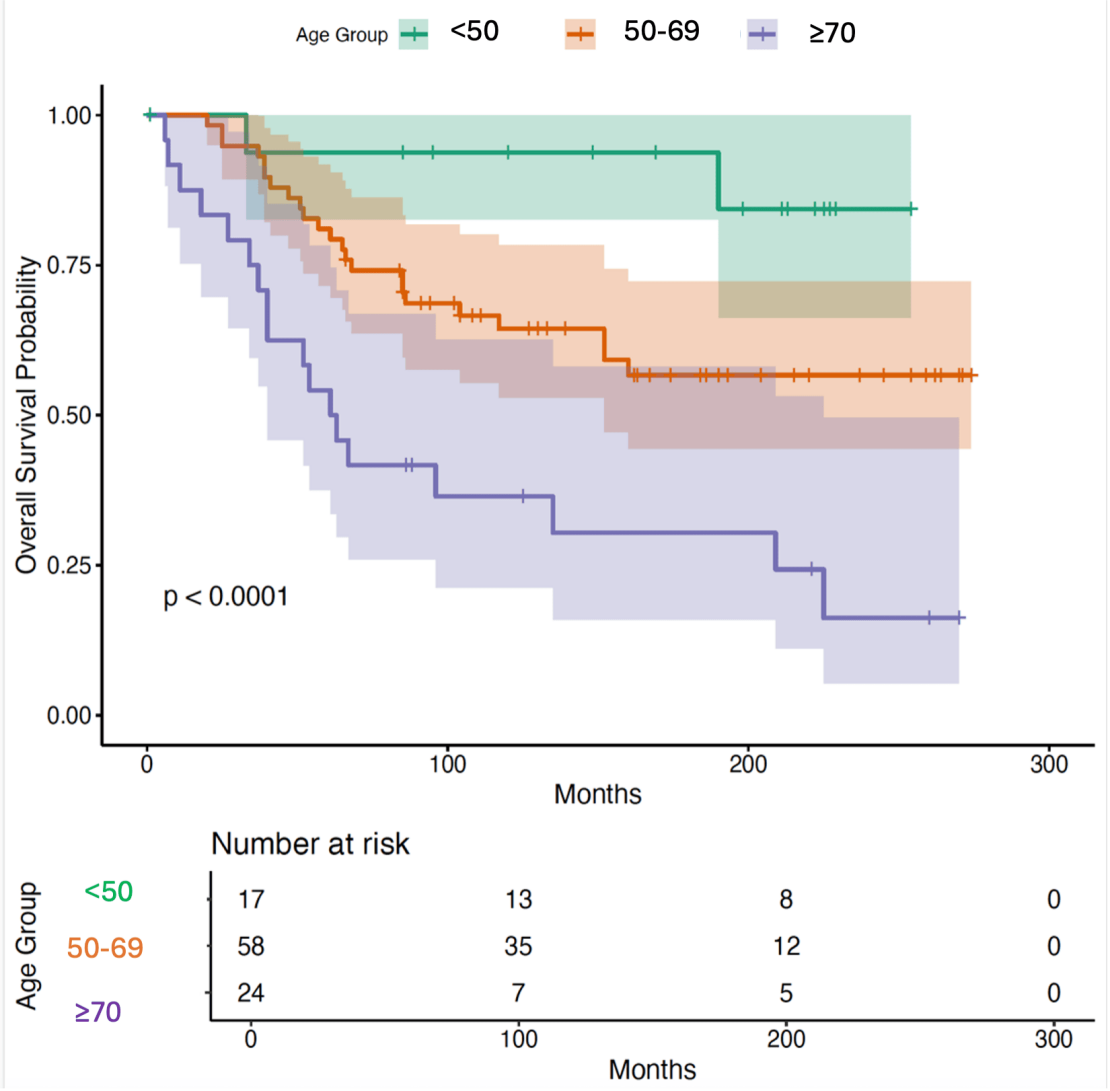
Relationship between age and survival Analysis demonstrated a significant difference in overall survival according to age. Patients <50 years old had the most favourable outcome, with relatively stable survival over the follow-up period. Survival declined more steeply among those aged 50-69 years, and most markedly for those above 70 years old. Data were obtained from the Surveillance, Epidemiology, and End Results (SEER) database, which uses standardized coding based on the International Classification of Diseases for Oncology, 3rd Edition, and SEER Summary Staging Manual [9,11,12].  Survival curves were generated using the Kaplan-Meier method, with the survminer package in R (R Foundation for Statistical Computing, Vienna, Austria) [13,18]. Differences between groups were tested with the log-rank test [14].

When survival was stratified by age, clear differences between patients emerged. Kaplan-Meier estimates revealed progressively worse survival with advancing age (log-rank χ² = 19.5, df = 2, p < 0.001). Patients under 50 years showed the most favourable outcomes, maintaining high survival probabilities throughout the follow-up interval. However, survival dropped sharply among patients aged 70 years and older, whilst those between 50 and 69 years displayed an intermediate pattern (Figure 1).

The multivariable Cox regression model reinforced the prognostic role of the age of patients. Compared with patients younger than 50 years, those aged 50-69 years had a hazard ratio (HR) of 4.00 (95% CI: 0.94-17.01, p = 0.061), suggesting a potential increase in risk, although this was not statistically significant. The 70 years and older age group carried the most significant risk, with an HR of 10.65 (95% CI: 2.46-46.07, p = 0.002). Thus, older age strongly predicted poorer survival.

Sex and survival

**Figure 2:**
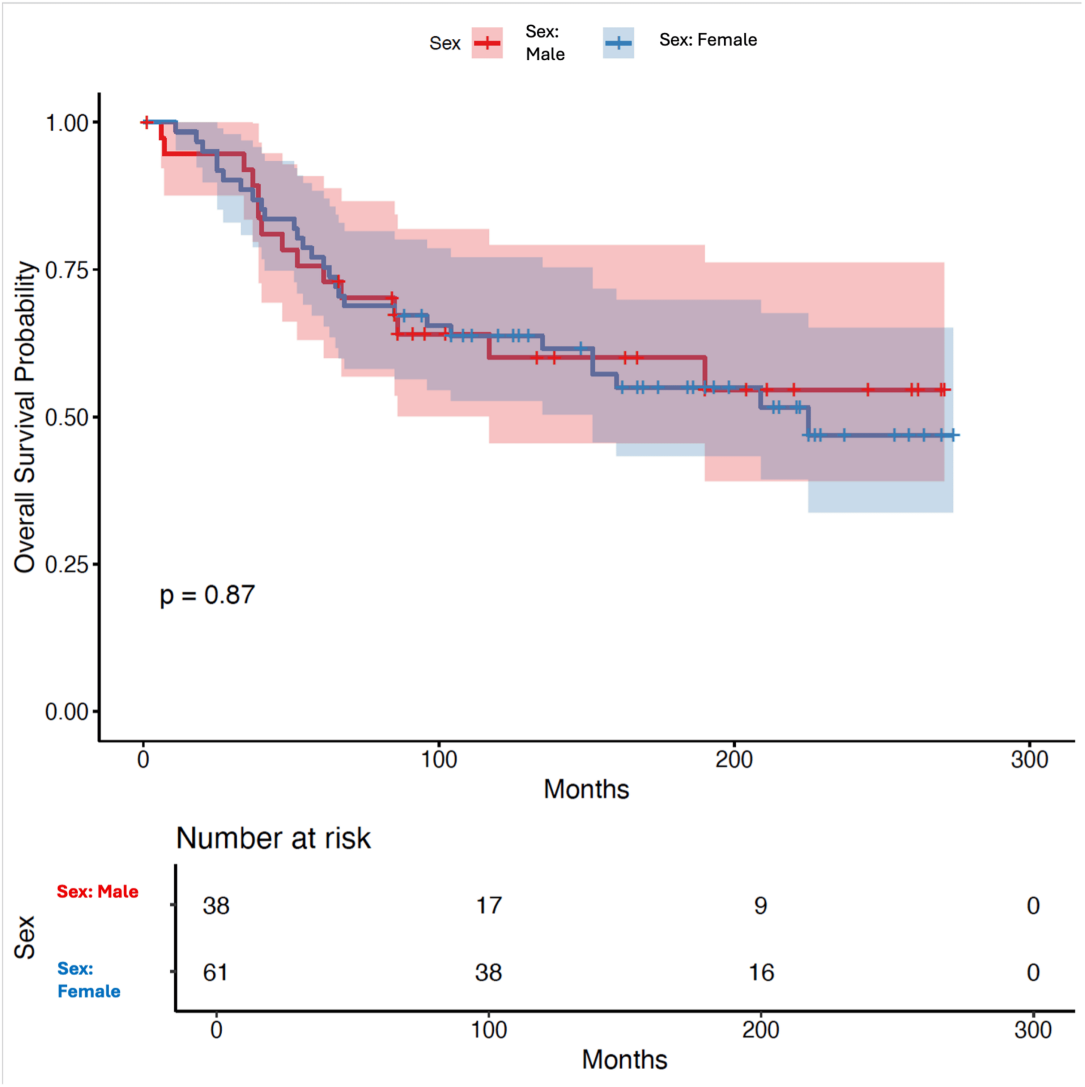
Relationship between sex and survival Survival analysis showed no significant difference in overall survival between male and female patients. Both groups demonstrated similar survival trajectories over time. Data were obtained from the Surveillance, Epidemiology, and End Results (SEER) database, which uses standardised coding based on the International Classification of Diseases for Oncology, 3rd Edition, and SEER Summary Staging Manual [9,11,12]. Survival curves were generated using the Kaplan-Meier method, with the survminer package in R (R Foundation for Statistical Computing, Vienna, Austria) [13,18]. Differences between groups were tested with the log-rank test [14].

By contrast, survival outcomes did not differ by the sex of patients. Kaplan-Meier analysis showed nearly identical curves for men and women (log-rank χ² = 0, df = 1, p = 0.87), with long-term survival remaining comparable between both groups (Figure 2). In the multivariable model, female sex had an HR of 1.02 (95% CI: 0.54-1.92, p = 0.944), confirming that sex was not a significant determinant of survival.

Model assessment

The Cox model demonstrated a concordance index of 0.68 (SE = 0.039), which indicates a moderate discriminatory ability. Likelihood ratio, Wald, and score tests were all highly significant (p < 0.01), hence supporting the robustness of age as a prognostic factor. Collectively, these findings highlight that patient age is a major predictor of survival in localised anterior uveal melanoma, whereas sex does not appear to influence outcomes in this cohort.

## Discussion

This study highlights the importance of age as a prognostic factor in iris and ciliary body melanoma, which has been treated with eye-preserving approaches. Older patients, particularly those aged 70 years or above, had significantly worse overall survival compared with younger individuals. In contrast, patient sex was not associated with survival, suggesting that demographic risk in this context is primarily driven by age.

The effect of age was both statistically strong and clinically significant. Patients ≥70 years faced more than a tenfold increase in mortality risk compared to those under 50 years, a finding consistent across both univariate and multivariable analyses. The 50-69-year age group also demonstrated a higher hazard of death, although the association narrowly missed statistical significance. The wide confidence intervals observed likely reflect smaller subgroup numbers rather than a true absence of effect. Overall, these findings point to a gradient of worsening prognosis with advancing age.

Several explanations may account for the negative influence of age. Declining immune surveillance in older individuals may permit the progression of micro metastatic disease [19]. Older patients are also more likely to have several comorbidities, which affect survival irrespective of melanoma [20]. Another possibility is that the biology of the tumour differs by age, with genetic or cytogenetic aberrations more prevalent in older patients. For example, in posterior uveal melanoma, unfavourable features such as monosomy 3 and *BAP1* mutations are more common with advancing age [21,22]. Whilst such data are not available in SEER, it is plausible that similar biological mechanisms are at play within anterior tumours.

The absence of sex-related survival differences in this study is notable. Previous research has suggested that hormonal or immunological factors might confer a survival advantage to women [5]. Some large registry analyses of posterior uveal melanoma have reported worse outcomes in men, although results are not consistent across all populations [23]. In this cohort of iris and ciliary body melanomas, survival was virtually identical between sexes, both on unadjusted analysis and in the multivariable models. This indicates that sex does not significantly affect prognosis in anterior uveal melanoma treated with conservative approaches.

Clinically, these findings underscore the importance of age as a readily available prognostic marker. Whilst molecular and histopathologic variables undoubtedly provide more refined prognostic information, they are not always accessible in routine practice or population-level registries. Awareness of the increased mortality risk in older patients may inform patient counselling, follow-up scheduling, and decisions around treatment intensity. Although eye-preserving treatment achieves excellent local tumour control, systemic survival remains strongly shaped by these demographic risk factors.

Finally, the moderate concordance index of the Cox model indicates that patients’ age provides meaningful, but not comprehensive, prognostic information. Hence, incorporation of molecular or pathological data would likely improve predictive accuracy. Nevertheless, in the absence of such detail, age remains a powerful and clinically relevant determinant.

The prognostic influence of age in uveal melanoma has been consistently reported in the literature. Mahendraraj et al., in a large SEER-based analysis of over 7,500 patients, identified increasing age as an independent predictor of poorer survival [24]. Similarly, Alfaar and colleagues, in a nationwide German cohort, found that patients over 60 years experienced worse outcomes [25]. Our results extend these findings to the subset of iris and ciliary body melanomas, confirming that the relationship between age and prognosis is not limited to posterior tumours but applies across different anatomical subgroups as well.

The question of sex-based differences in survival has been less clear-cut, however. A recent long-term review by Stålhammar suggested a modest survival advantage for women in posterior uveal melanoma, which potentially reflects hormonal or immune-mediated factors [5]. Other large datasets, such as those reported by Weinberger et al., have not reproduced this effect, with sex showing no significant impact after multivariable adjustment [7]. Our findings are consistent with the latter, showing no measurable difference between men and women. This suggests that any sex effect is either weak, inconsistent across populations, or absent within anterior tumour sites.

Studies focusing specifically on iris and ciliary body melanomas are relatively limited. Sabazade et al. observed more favourable outcomes for iris melanoma compared with small choroidal melanomas, although they also noted the influence of age on prognosis [26]. Our data, derived from a larger SEER cohort, confirms the detrimental role of advanced age in this subset and provides stronger statistical evidence. The rarity of these tumours underscores the value of population-based registries in generating insights that single-institution studies cannot reliably provide.

Our study also reflects broader epidemiologic trends in treatment. The declining use of enucleation and rising adoption of radiotherapy or local resection have shaped cancer management over recent decades. Swedish registry data suggest that survival may have improved due to earlier diagnosis and more widespread use of brachytherapy [8]. In contrast, SEER-based analyses generally do not demonstrate substantial gains in long-term survival, highlighting that demographic and biological factors remain key determinants of outcome [27]. Our results, restricted to patients treated with eye-preserving strategies, suggest that although local control is excellent, systemic prognosis is still primarily influenced by patient age. It is essential to note that treatment paradigms would have continued to evolve during the period of the study (2000-2017), with plaque brachytherapy and proton beam therapy becoming more widely adopted, whilst enucleation has declined [28]. Although these eye-preserving approaches provide excellent local control, they have not always translated into improved survival in SEER analyses, perhaps due to the predominant influence of age and tumour biology [29]. This evolution emphasises the need to interpret survival outcomes in the context of shifting therapeutic practices. 

Limitations

This study has several limitations, primarily related to the use of population-based SEER data. Key prognostic variables, such as the thickness of the tumour, the largest basal diameter, histopathological features, and cytogenetic alterations (e.g., monosomy 3, *BAP1* status), are not available in SEER and could not be incorporated. Furthermore, site coding within SEER may not always clearly distinguish iris from ciliary body melanoma, raising potential for misclassification [30]. Additionally, information on radiation technique, dosage, and surgical margin status is unavailable, which can limit the ability to evaluate treatment-related factors that may influence survival. Study participants who received chemotherapy were selected for this intervention by the lead clinician, which may affect the systematic recording of the intervention by SEER.

SEER also does not provide detailed data on systemic therapies, adjuvant treatments, or comorbidities, which may confound survival outcomes. The retrospective observational design also introduces the potential for selection bias. Finally, although the study period spans 2000-2017, advances in diagnostic and therapeutic approaches during this time may have influenced outcomes, and temporal trends could not be fully accounted for.

Despite these limitations, the large sample size and population-based design of SEER provide valuable insights into demographic prognostic factors in this rare malignancy, supporting the relevance of our findings to real-world clinical practice.

## Conclusions

In this population-based study of patients with iris and ciliary body melanoma treated with eye-preserving modalities, we identified that age was a strong prognostic factor for overall survival, whereas sex was not associated with outcomes. Kaplan-Meier analysis and log-rank testing demonstrated significantly worse survival among patients aged ≥70 years old, whilst those under 50 had the most favourable outcomes. These findings were reinforced by multivariable Cox regression, where an age of 70 years or over was associated with more than a tenfold increased risk of death compared with patients younger than 50. By contrast, we observed no meaningful difference in survival between men and women.

Our results highlight the prognostic importance of patient age in localised anterior uveal melanoma, consistent with broader trends observed in posterior uveal melanoma and other malignancies. The absence of sex-related differences suggests that, in this specific cohort, biological or hormonal influences may play a limited role in modifying survival. These findings carry practical implications for risk stratification and counselling of patients who are considering conservative treatment. Future research that incorporates tumour-specific biological data, such as cytogenetics or molecular profiling, is needed to refine prognostic models and improve personalised management in this rare but clinically significant subgroup.
